# Visualization and tracking of tubule-derived, fluorescent-labeled NS1 as a marker of bluetongue virus in living cells

**DOI:** 10.1128/jvi.00896-25

**Published:** 2025-08-07

**Authors:** Yuqing Song, Xuechun Liu, Yongjun Che, Jinlong Wang, Zhancheng Tian, Guiquan Guan, Polly Roy, Hong Yin, Junzheng Du

**Affiliations:** 1 State Key Laboratory for Animal Disease Control and Prevention, College of Veterinary Medicine, Lanzhou University, Lanzhou Veterinary Research Institute, Chinese Academy of Agricultural Sciences 111658 , Lanzhou, China; 2 Gansu Province Research Center for Basic Disciplines of Pathogen Biology, Lanzhou, China; 3 Department of Infection Biology, London School of Hygiene and Tropical Medicine, London, United Kingdom; 4 Jiangsu Co-innovation Center for Prevention and Control of Important Animal Infectious Diseases and Zoonoses, Yangzhou University 38043, Yangzhou, China; University of Michigan Medical School, Ann Arbor, Michigan, USA

**Keywords:** bluetongue virus, non-structural protein 1, reverse genetics, laser confocal live-cell imaging

## Abstract

Bluetongue virus (BTV) is an arbovirus (genus, *Orbivirus*; family, *Sedoreoviridae*) transmitted among ruminant hosts by *Culicoides* biting midges (genus, *Culicoides*). *Orbivirus sp*. produce large numbers of virus-specific tubules during infection. Non-structural protein 1 (NS1) derived from BTV is a highly conserved tubule-forming protein that exists in infected cells as tubular and non-tubular forms. However, the lack of tagged viruses has limited dynamic tracking of NS1 tubule protein levels in living cells. Here, four recombinant viruses (BTV1-NS1-156TC, BTV1-NS1-156eGFP, BTV1-NS1-534eGFP, and BTV1-NS1-552eGFP) were successfully rescued using a BTV reverse genetics strategy by introduction of biarsenical-tetracysteine or enhanced green fluorescent protein (eGFP) tags to the BTV NS1 protein. Inserting these tags at these three sites resulted in different equilibrium states of NS1 between the tubular and non-tubular forms. NS1 was tracked in live cells to observe the dynamic movement and aggregation processes of NS1 tubules. The formation of NS1 tubules is a prerequisite for NS1 aggregation, and NS1 tubules are dependent on cellular microtubules to aggregate at the microtubule organizing center and encapsulate by vimentin to form aggresomes. This study confirms the possibility of controlling the conversion ratio between NS1 monomers and tubule forms, for the first time elucidates the movement and aggregation processes of NS1 tubules by live-cell imaging, and confirms that NS1 tubules form aggresomes.

**IMPORTANCE:**

While extensive research has established BTV as a model system to study large non-enveloped viruses, critical gaps remain in our understanding of its biology. During infection, BTV NS1 assembles into abundant tubulars, a hallmark feature of the *Orbivirus* genus. In this study, fluorescently tagged BTV NS1 proteins were engineered, and four recombinant viruses were successfully rescued. Insertion of these tags altered the equilibrium between tubular and monomeric NS1 conformations, demonstrating the feasibility of modulating the transition ratio between structural states. Additionally, NS1 tubules depend on microtubule-mediated intracellular transport for aggregation and subsequent aggresome formation. These findings provide a basis for the application of NS1 in vaccine delivery, therapy, nanotechnology, and future explorations of BTV pathogenesis.

## INTRODUCTION

Bluetongue (BT), caused by the Bluetongue virus (BTV), is a non-contagious, insect-transmitted, viral disease of economic importance to both domestic and wild ruminants, especially sheep, goats, and cattle, which is spread by hematophagous biting midges of *Culicoides* spp. presenting as oral erosions and ulcers, lameness, face edema, and tongue cyanosis (1).

BTV (genus, *Orbivirus*; family, *Sedoreoviridae*) is a non-enveloped icosahedral capsid structure consisting of 10 dsRNA fragments encoding seven structural proteins (VP1–VP7) and five non-structural proteins (NS1, NS2, NS3/3A, NS4, and NS5) (2). These non-structural proteins play important roles in self-replication during BTV infection. Specifically, NS1 enhances viral protein synthesis (3), NS2 binds ssRNA and recruits structural proteins that form viral inclusion bodies (4), NS3 is involved in mature viral assembly and release (5), NS4 regulates innate immune responses and antagonizes both type I interferon signaling with NS3, thus enhancing BTV replication (6), and NS5 localizes to the nucleolus and then diffuses within the nucleus and cytoplasm, thereby preventing degradation of ribosomal RNA during infection and reducing host protein synthesis (2).

Viral infection of cells requires rapid hijacking of the cellular translational machinery for synthesis of viral proteins (7). Each *de novo* transcript of the rotavirus and BTV genomes is processed and modified to have a 5′ cap-I structure, but lacks a poly(A) tail (8). During rotavirus infection, the viral protein NSP3A binds to the 3′ end of viral mRNA and interacts with eIF4GI and expels the poly(A)-binding protein from eIF4F (9, 10). BTV NS1 specifically binds to the 3′ terminal sequence of BTV mRNA and upregulates viral protein synthesis (3). NS1, the most highly expressed viral protein, forms a number of characteristic tubules in infected cells during BTV replication (11). Mature viral particles have been observed on NS1 tubular structures by transmission electron microscopy. NS1-specific monoclonal antibody reacts with BTV particles in the vicinity of inclusion bodies or upon egression from infected cells (12), suggesting that NS1 may play a role in the transfer of BTV particles to the plasma membrane. Expression of a recombinant single-chain antibody fragment against NS1 in BSR cells, followed by inoculation with BTV, has been shown to delay the cytopathic effect (CPE) and shift from lytic to non-lytic release of BTV (13). Although it is thought that the NS1 protein aggregates around the nucleus of BTV-infected cells, the dynamics of this aggregation process have not yet been fully elucidated.

BTV reverse genetics has provided the basis for fluorescent labeling of viral proteins, which is a powerful tool to study the processes of virus-host interactions, antiviral medication screening, neutralizing antibody detection, *in vivo* viral infection, and host immunity by real-time fluorescence imaging of viral proteins in living cells (14–16). Insertion of exogenous genes is potentially lethal to viruses and is particularly difficult for segmented double-stranded RNA viruses, such as BTV. Analysis of RNA-RNA and RNA-structural protein interactions demonstrated that the BTV genome is comprised of 10 dsRNA segments encapsulated by two concentric protein shells (17–19). In addition to impairing RNA-RNA interactions, insertion of foreign gene fragments may also affect the functions of viral proteins, which could hinder or even stop genome packaging and viral infectivity, resulting in a lower viral yield or rescue failure (20). In this study, we successfully rescued recombinant BTVs expressing biarsenical-tetracysteine (TC) or enhanced green fluorescent protein (eGFP) tags at three different sites of NS1 of different recombinant BTVs in different equilibrium states of non-tubular and tubular formation. Subsequently, the movement of NS1 tubule proteins gradually aggregated around the nucleus, as observed by laser confocal live-cell imaging. The formation of NS1 tubules is a prerequisite for NS1 aggregation. Nocodazole, a microtubule inhibitor, inhibited aggregation of NS1 proteins, indicating that trafficking of NS1 is dependent on cellular microtubule transport. Furthermore, NS1 proteins aggregated in the microtubule organizing center (MTOC) of cells and were wrapped in cage-like structures formed by vimentin to form an aggresome.

## RESULTS

### Selection of insertable foreign gene sites of NS1

**Fig 1:**
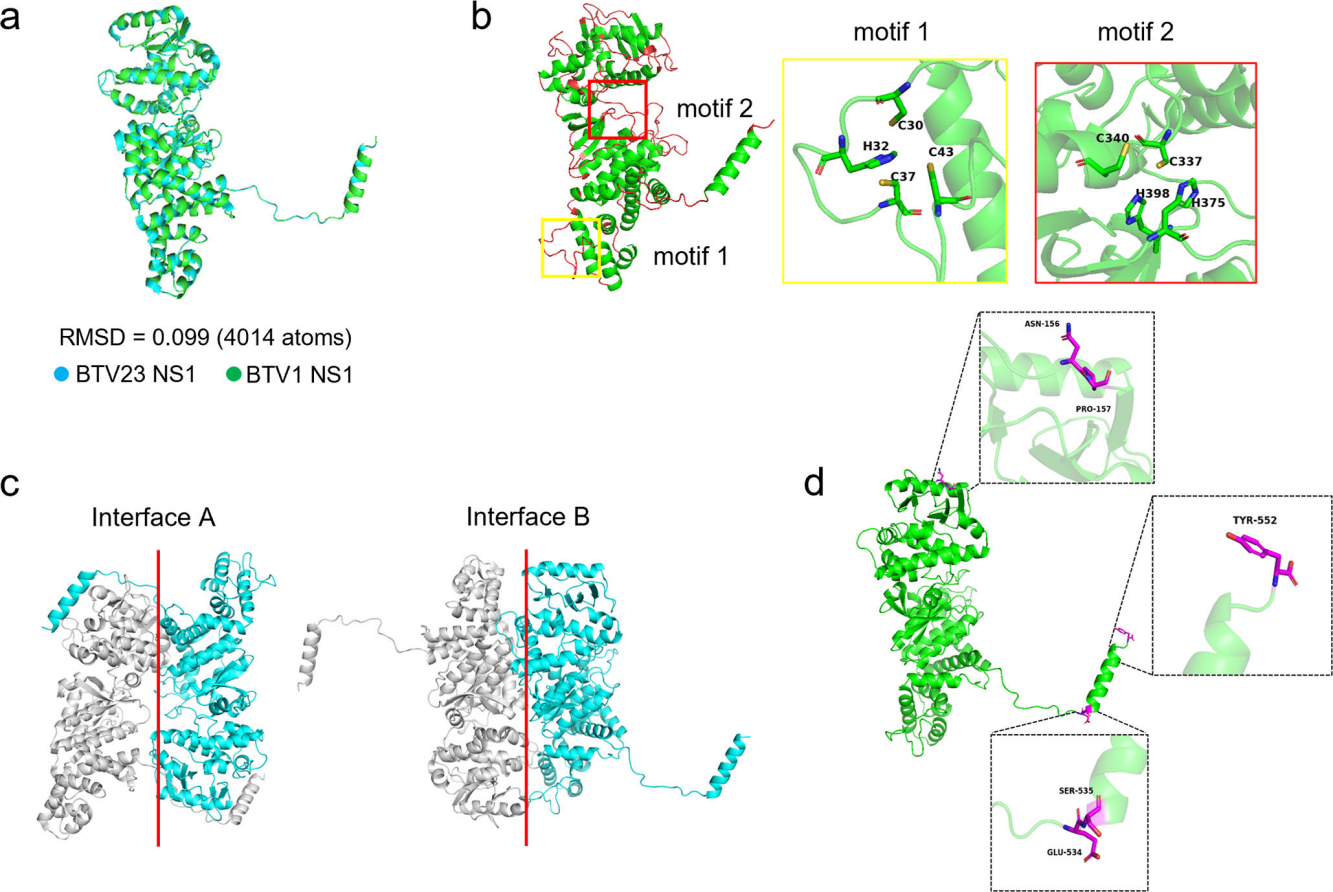
Selection of insertable sites for NS1. (**a**) Alphafold 2 software was used to predict the structure of BTV1 NS1 and conduct comparisons with the resolved BTV23 NS1. (**b**) The random coiled regions (red) and motif locations of BTV1 NS1 are shown. (**c**) NS1 of interfaces A (left) and B (right) is shown (22). (**d**) Diagrammatic representation of the insertion sites of exogenous genes.

Insertion of the expressed fluorescent protein at different sites may have different effects on the growth characteristics of viruses. The random coiled region exposed to the protein surface is thought to be a more likely insertion site for foreign sequences. The atomic structure of BTV23 NS1 has been resolved, and the amino acid sequence shares 98.4% similarity with that of BTV1 NS1. The structure of BTV1 NS1 predicted with AlphaFold 2 software was very similar to that of BTV23 NS1 (both, root mean square deviation = 0.099) (Fig. 1a) (21). The structure included random coiled regions (Fig. 1b), which greatly facilitated the search for potential insertion sites.

First, the random coiled regions within the protein interior were excluded. Second, the NS1 monomer contained two zinc-finger-like motifs, and mutations of either motif failed to rescue the virus in reverse genetics experiments (Fig. 1b), suggesting that these motifs may have important biological functions. Therefore, regions related to the two motifs were excluded. To avoid an effect on NS1 tubule formation as much as possible, potential insertion sites were designed to avoid interfaces A and B and junctions of different domains (Fig. 1c). Ultimately, based on the above analysis, three regions (between amino acid residues 156 and 157, 534 and 535, as well as at the C-terminus of NS1) can be exploited to introduce tags (Fig. 1d).

### Generation of recombinant fluorescent viruses

**Fig 2:**
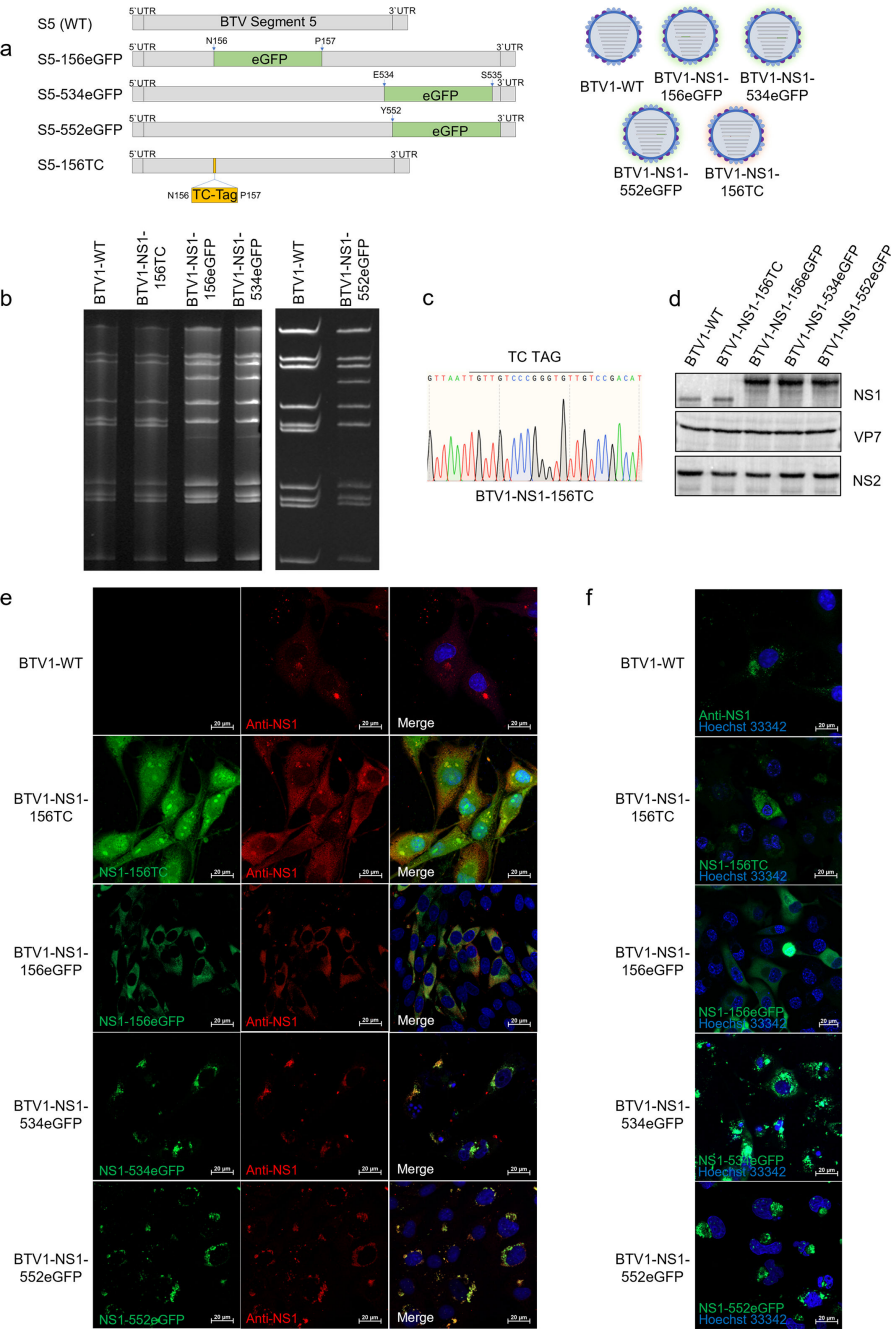
Rescue of recombinant viruses with eGFP or TC tags. (**a**) Diagrammatic representation of the rescued recombinant viruses. eGFP or TC tags were inserted between amino acid residues 156 and 157, 534 and 535, or the C terminus of the NS1 protein encoded by BTV S5, and the recombinant viruses were successfully rescued by reverse genetics. (**b**) Genomic dsRNAs from BSR cells infected with BTV1-WT, BTV1-NS1-156TC, BTV1-NS1-156eGFP, BTV1-NS1-534eGFP, and BTV1-NS1-552eGFP were purified and analyzed on a nondenaturing polyacrylamide gel. (**c**) Sequence electropherograms of S5 RT-PCR products from the TC-tagged virus. The positions of the TC tags are indicated above each panel. (**d**) Analysis of protein expression. BSR cells were infected with BTV1-WT and recombinant viruses. Specific antibodies against NS1, VP7, and NS2 were used to detect viral protein expression. BSR cells were infected with BTV1-WT and recombinant viruses (**e and f**). NS1 was detected by using the rabbit anti-NS1 polyclonal antibody (red) (**e**). The eGFP tag was directly presented in green, and the TC tag was visualized in green using a tetra-cysteine labeling detection kit (**e and f**). Nuclei were stained with Hoechst 33342 (blue) (**e and f**). Bar, 20 µm.

An eGFP tag was inserted between amino acid residues 156 and 157, 534 and 535, or the C terminus of NS1, and a TC tag was inserted between amino acid residues 156 and 157 (Fig. 2a). Capped S5 T7 transcripts (BTV1-S5 and the fusion-tagged S5) were generated to recover fluorescently labeled viruses. The reverse genetics system used nine T7-derived RNA transcripts (S1–S4 and S6–S10) to transfect BSR cells along with the WT S5 segment or S5-156TC, S5-156eGFP, S5-534eGFP, and S5-552eGFP transcripts. As with BTV1-WT, BTV1-NS1-156TC, BTV1-NS1-156eGFP, BTV1-NS1-534eGFP, and BTV1-NS1-552eGFP were successfully rescued. Viral genome dsRNA was extracted from infected cells, purified, and analyzed on non-denaturing polyacrylamide gels (Fig. 2b). The results showed that the S5 segments of BTV1-NS1-156eGFP, BTV1-NS1-534eGFP, and BTV1-NS1-552eGFP migrated at a slower rate than BTV1-WT due to the presence of the eGFP coding sequence, confirming incorporation of the modified S5 segment into the BTV-1 genome (Fig. 2b). Other than the insertion of the TC tag, there was no significant difference in the S5 segments of BTV1-NS1-156 TC and BTV1-WT (Fig. 2c). Sequencing of the S5 segments of other recombinant viruses confirmed correct incorporation of the eGFP gene. The NS1 proteins of BTV1-WT and BTV1-NS1-156TC were 64 kDa, while BTV1-NS1-156eGFP, BTV1-NS1-534eGFP, and BTV1-NS1-552eGFP were approximately 91 kDa (Fig. 2d). The VP7 and NS2 proteins of the recombinant viruses and BTV1-WT were indistinguishable (Fig. 2d). BSR cells infected with BTV1-WT and recombinant viruses were incubated with the NS1 antibody and labeled with the TC dye. Red fluorescence of labeled NS1 and green fluorescence of eGFP or TC were colocalized (Fig. 2e). In summary, the eGFP or TC tags were successfully inserted at the corresponding positions, and the recombinant viruses were rescued.

BTV1-WT and recombinant viruses were observed by confocal microscopy (Fig. 2f). Before incubation with a polyclonal antibody against NS1 and fluorescence labeling with a fluorescent secondary antibody, BSR cells were infected with BTV1-WT and fluorescence was observed by laser confocal microscopy. BSR cells were directly observed after infection with BTV1-NS1-156eGFP, BTV1-NS1-534eGFP, and BTV1-NS1-552eGFP. BSR cells were also observed after infection with BTV1-NS1-156TC using TC dye fluorescence labeling. The results showed that NS1 of BTV1-NS1-534eGFP and BTV1-NS1-552eGFP accumulated around the nucleus, similar to BTV1-WT (Fig. 2f). In contrast, NS1 of BTV1-NS1-156TC and BTV1-NS1-156eGFP was diffusely distributed throughout the cytoplasm (Fig. 2f), indicating disrupted NS1 tubule formation.

### Characterization of recombinant fluorescent viruses

**Fig 3:**
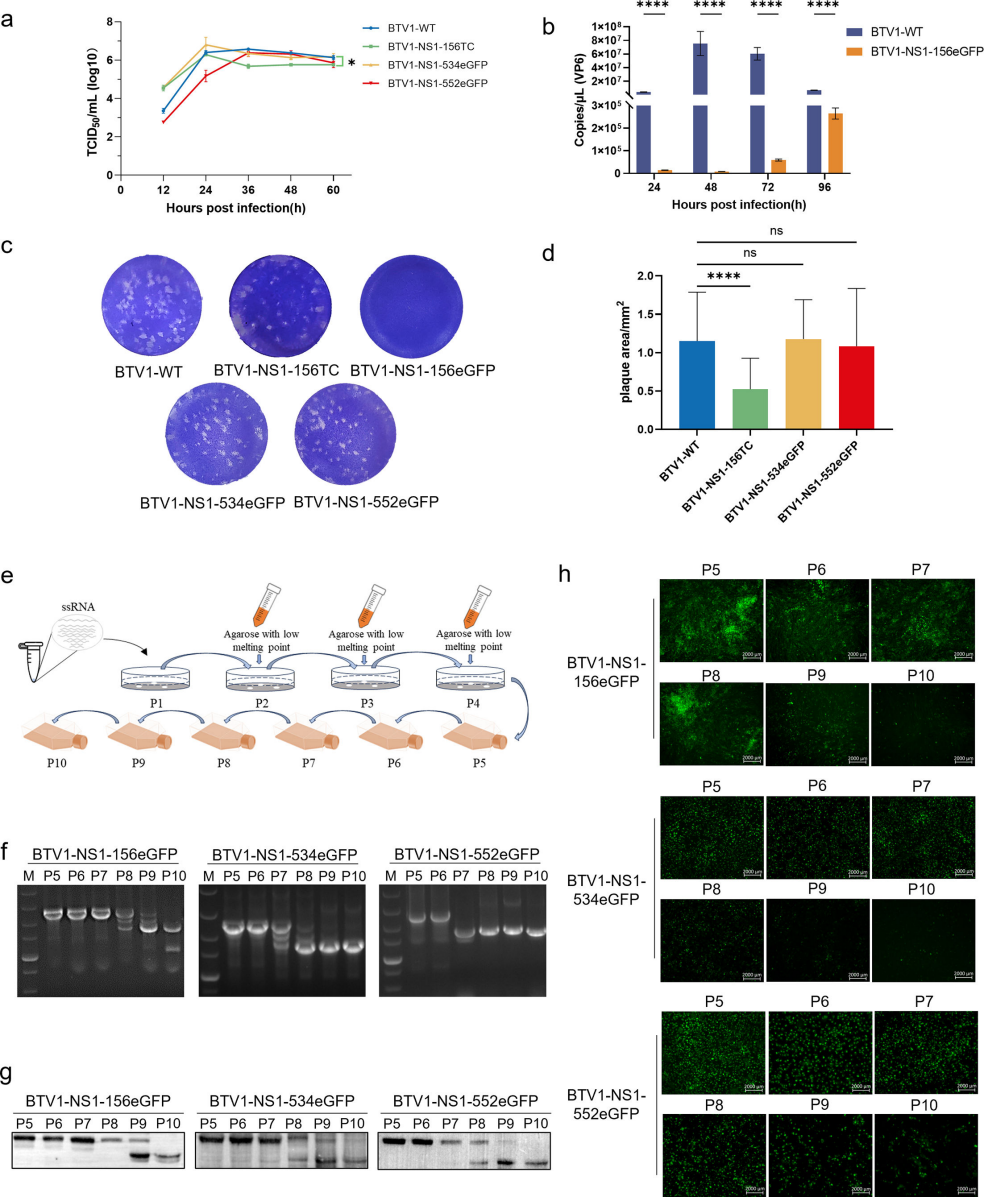
Growth characteristics and genetic stability of the recombinant viruses *in vitro*. (**a**) Viral replication kinetics of viruses in BSR cells. Confluent monolayers were infected with the virus (MOI = 0.1), and the virus titers were determined as described in Materials and Methods. All assays were performed in triplicate (*n* = 3). An asterisk indicates that the titers of BTV1-NS1-156TC at 60 h post-infection are statistically significantly lower than those of BTV1-WT (*P* < 0.05). (**b**) Copies (VP6) of BTV1-NS1-156eGFP and BTV1-WT in BSR cells. Confluent monolayers were infected with the virus (MOI = 0.001), and the virus titers were determined (*n* = 3). ****P < 0.0001. (**c**) Plaque phenotype. Representative pictures of viral plaques in BSR cells (24-well plate format) infected with the indicated viruses are shown. (**d**) Statistical analysis of the viral plaque area. Monolayers of BSR cells were inoculated with viruses. Following infection for an identical duration, the cells were stained with crystal violet and photographed. The plaque areas were quantified using ImageJ software. ****P < 0.0001. (**e**) Diagrammatic representation of the virus purification process. The virus was rescued by transfecting T7 BTV transcripts (P1), and then the reservoir was obtained by monocloning three successive times (P2, P3, and P4). Finally, the reservoir was passaged to P10. Viruses from different passages were collected to amplify S5 by RT-PCR (**f**), to measure NS1 protein size by Western blot analysis (**g**), and to visualize eGFP by fluorescence microscopy (**h**).

BSR cells were infected at an MOI of 0.1, and viral growth was monitored over 60 h. The growth curves of BTV1-NS1-534eGFP and BTV1-NS1-552eGFP in BSR cells were similar to those of BTV1-WT (Fig. 3a). The viral titer harvested by BTV1-NS1-156eGFP only reached 2.86 × 10^4^ TCID_50_/mL, which did not allow for generation of a growth curve. BSR cells were infected at an MOI of 0.001 to determine the level of VP6 RNA of BTV1-NS1-156eGFP and BTV1-WT (Fig. 3b). The results showed that insertion of the eGFP tag between amino acid residues 156 and 157 severely attenuated viral replication. The successful rescue of BTV1-NS1-156eGFP confirmed that foreign genes can be inserted between amino acids 156 and 157 of NS1, although the large size of the eGFP gene may affect packaging of the viral genome or the function of NS1. Therefore, BTV1-NS1-156TC was rescued by inserting a TC tag between amino acid residues 156 and 157 of NS1. BTV1-NS1-156TC exhibited slightly lower growth than BTV1-WT (Fig. 3a).

Plaque analysis of the recombinant viruses showed that BTV1-NS1-156TC, BTV1-NS1-534eGFP, and BTV1-NS1-552eGFP were able to form obvious plaque morphologies, similar to BTV1-WT (Fig. 3c). BTV1-NS1-156eGFP could not form plaques, likely due to the severely reduced replication capacity. The plaque size was very heterogeneous among these viruses. However, plaques generated by BTV1-NS1-156TC were significantly smaller than those generated by BTV1-WT, BTV1-NS1-534eGFP, and BTV1-NS1-552eGFP (0.52 ± 0.4 vs 1.15 ± 0.63, 1.18 ± 0.51, and 1.08 ± 0.75 mm^2^, respectively) (Fig. 3d). These results indicate that between amino acid residues 156 and 157, 534 and 535, or the C terminus of NS1 are usable insertion sites for the heterologous tag. However, insertion of the eGFP tag between amino acid residues 156 and 157 severely attenuated virus replication and plaque formation.

The rescued P1 generation viruses were purified by monocloning three successive times and then consecutively passed on to the P10 generation (Fig. 3e). The genetic stability of BTV1-NS1-156eGFP, BTV1-NS1-534eGFP, and BTV1-NS1-552eGFP was assessed by RT-PCR (Fig. 3f), Western blot analysis (Fig. 3g), and fluorescence microscopy (Fig. 3h). As shown by RT-PCR, BTV1-NS1-156eGFP could be stably inherited over seven generations, and BTV1-NS1-534eGFP and BTV1-NS1-552eGFP could be stably inherited over six generations. However, BTV1-NS1-156eGFP, BTV1-NS1-534eGFP, and BTV1-NS1-552eGFP all produced recombinant NS1 bands and obvious fluorescence within the P8 generation. Total RNA was extracted from BTV1-NS1-156TC-infected cells for RT-PCR analysis and sequenced. The NS1 sequencing results of the tenth generation of BTV1-NS1-156TC showed that the TC tag still existed, indicating that the BTV1 TC tag could be stably inherited.

### Effect of tag insertion on NS1 tubule formation

**Fig 4:**
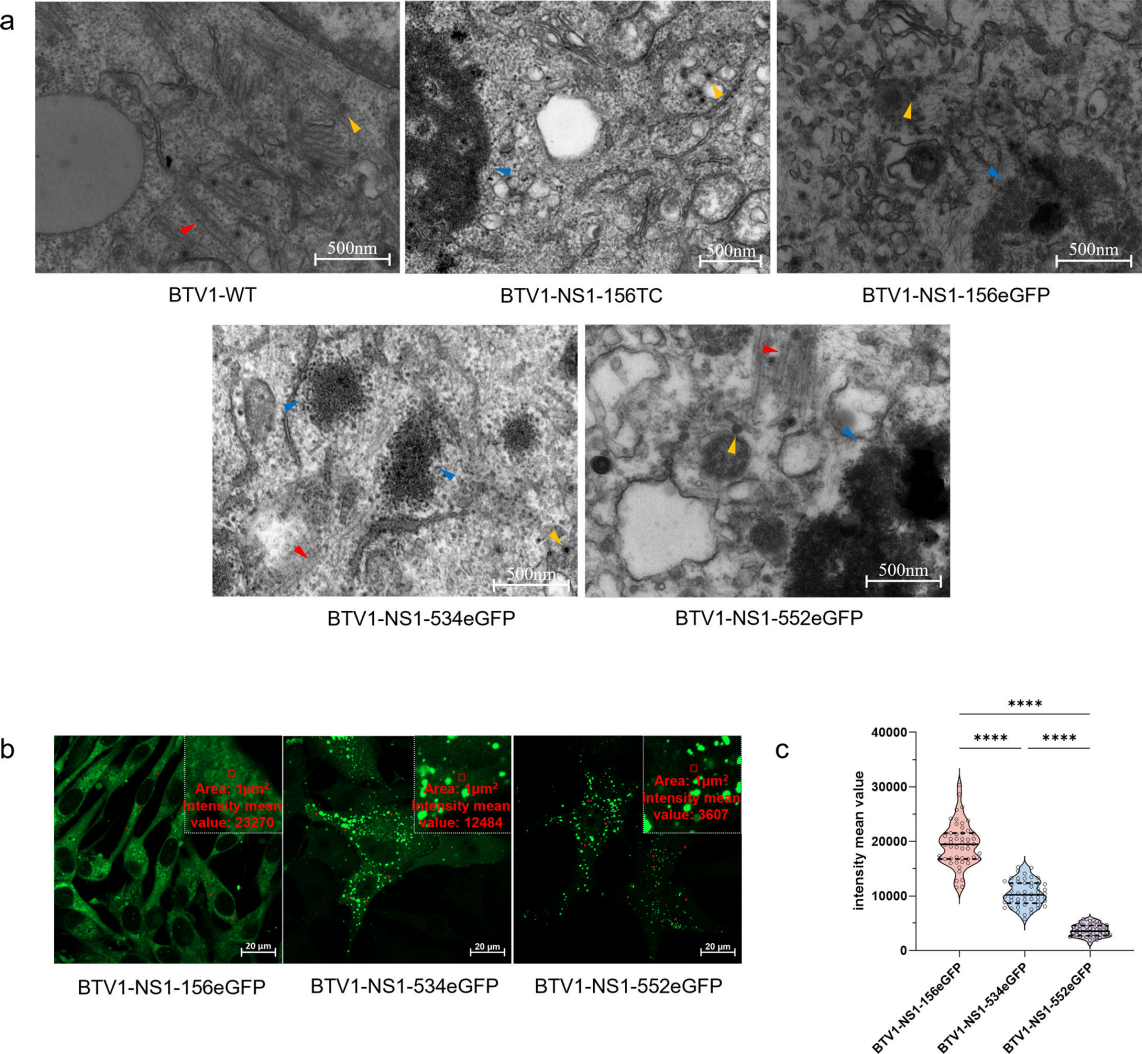
Effect of tag insertion on NS1 tubules. (**a**) BSR cell monolayers were infected with BTV1-WT and recombinant viruses. Before CPE of BSR cells, the cells were fixed for 24 h and then processed for transmission electron microscopy. Red triangles indicate NS1 tubules, orange triangles indicate BTV particles, and blue triangles indicate viral inclusion bodies. Bars, 500 nm. (**b**) Representative pictures of BSR cells infected with BTV1-NS1-156eGFP, BTV1-NS1-534eGFP, and BTV1-NS1-552eGFP. Higher magnification is shown in the upper insets. The intensity mean value in the box in red at 1 µm^2^ was measured. (**c**) Statistical analysis of the green fluorescence background of BTV1-NS1-156eGFP, BTV1-NS1-534eGFP, and BTV1-NS1-552eGFP at three different positions of 50 cells (*n* = 50) was quantified under the same shooting parameters, as indicated by the red dots in (**a**). ****P < 0.0001.

It has been reported that replacing key amino acids at the C-terminus of NS1 does not interfere with recovery of the recombinant virus, but NS1 loses the ability to form NS1 tubules (22). To determine whether tag insertion actually disrupts tubule formation, BSR cells infected with the recombinant viruses and BTV1-WT were collected and ultrathin sections were made, before the production of CPE, which were observed by transmission electron microscopy (Fig. 4a). The results showed that, identical to BTV1-WT, BTV1-NS1-534eGFP and BTV1-NS1-552eGFP formed a large number of NS1 tubules in BSR cells, while BTV1-NS1-156TC and BTV1-NS1-156eGFP did not, consistent with the results presented in Fig. 2f.

Intriguingly, BTV1-NS1-156eGFP, BTV1-NS1-534eGFP, and BTV1-NS1-552eGFP had different green fluorescent backgrounds under the same shooting parameters (Fig. 4b). Due to the weak fluorescent background of the TC dye, BTV1-NS1-156TC was not included for comparison. The fluorescence intensity of the background in three random regions of the cytoplasm of 50 cells was quantified, which revealed significant differences in the fluorescence background values of the three recombinant viruses (Fig. 4c). NS1 of BTV1-NS1-156eGFP did not form tubules, was scattered throughout the cell, and had the highest fluorescence background. The green fluorescent background of BTV1-NS1-534eGFP was more pronounced in the cytoplasm than BTV1-NS1-552eGFP under the same shooting parameters, suggesting that insertion of the eGFP tag between amino acid residues 534 and 535 has affected the dynamic balance between NS1 monomers and the formation of NS1 tubules.

### NS1 tubules accumulate around the nucleus via microtubules

**Fig 5:**
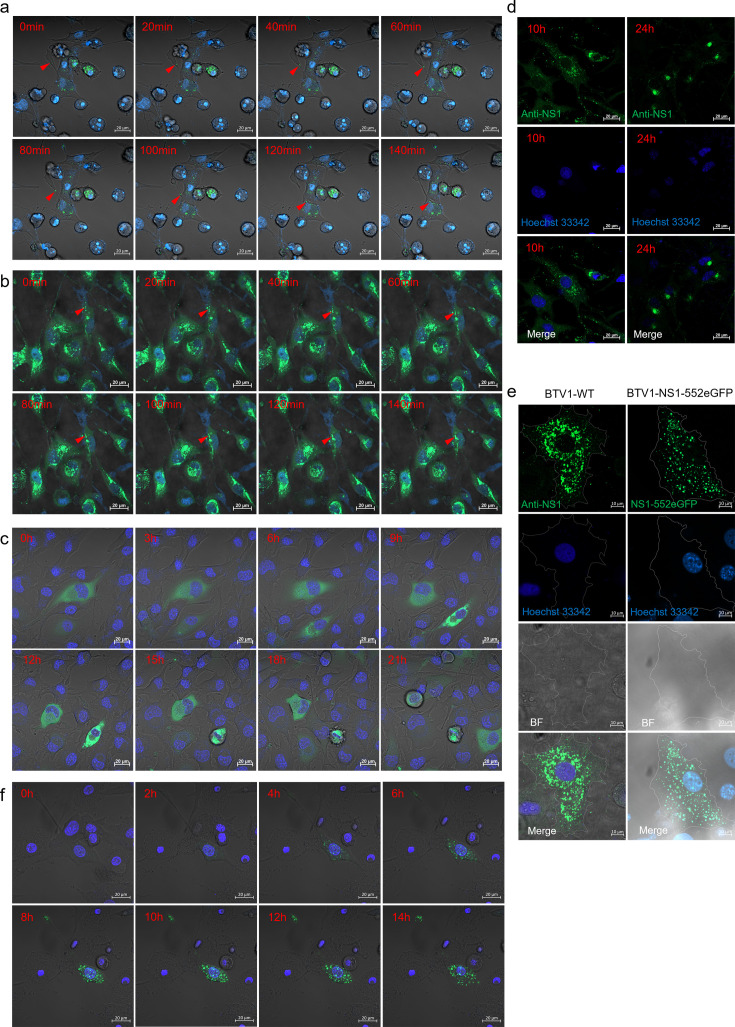
NS1 moves through microtubules and accumulates on the side of the nucleus. (**a, b, and c**) Live-cell confocal tracking of BTV1-NS1-552eGFP (**a**), BTV1-NS1-534eGFP (**b**), and BTV1-NS1-156eGFP (**c**) in BSR cells. The red triangle indicates movement of NS1 to the nucleus. Bars, 20 µm. (**d**) BSR cells were initially infected with BTV1-WT (MOI = 0.1) for 10 and 24 h. BTV1-WT was detected for NS1 using the rabbit anti-NS1 polyclonal antibody. Nuclei were stained with Hoechst 33342 (blue). Bar, 20 µm. (**e**) BSR cells were infected with BTV1-NS1-552eGFP and BTV1-WT for 12 h and then cultured for another 12 h after adding the microtubule inhibitor nocodazole (final concentration, 500 ng/mL). The cells were then fixed and BTV1-WT was tested using rabbit polyclonal antibody against NS1. Nuclei were stained with Hoechst 33342 (blue). Bar, 10 µm. (**f**) Live-cell confocal tracking of BTV1-NS1-552eGFP in BSR cells after nocodazole treatment. Bar, 20 µm.

Previously, a large number of electron microscopy images demonstrated that NS1 tubules had localized around the nucleus, but the dynamic process of aggregation remains unclear. Live-cell imaging was utilized to observe BSR cells infected with BTV1-NS1-156eGFP, BTV1-NS1-534eGFP, and BTV1-NS1-552eGFP, with images captured every 20 min. The results showed that during the early stages of infection, NS1 tubules of BTV1-NS1-534eGFP and BTV1-NS1-552eGFP were randomly distributed in the cytoplasm. However, as infection progressed, these tubules gradually clustered near the nucleus (Fig. 5a and b; Videos S1 and S2). In contrast, the fluorescence intensity of BTV1-NS1-156eGFP increased over time without aggregation (Fig. 5c; Video S3). Immunofluorescence analysis of BSR cells infected with BTV1-WT at 10 h and 24 h post-infection (Fig. 5d) revealed a similar transition of NS1 from the random distribution to perinuclear clustering, mirroring the behavior of BTV1-NS1-534eGFP and BTV1-NS1-552eGFP. These findings indicate that NS1 undergoes a dynamic aggregation process, dependent on the formation of NS1 tubules.

To investigate the molecular mechanism underlying the movement of NS1 tubules, BSR cells were infected with BTV1-WT and BTV1-NS1-552eGFP for 12 h and then treated with the microtubule inhibitor nocodazole. The laser confocal microscopy results showed that NS1 tubules were no longer clustered on the side of the nucleus but rather scattered around the nucleus (Fig. 5e), as confirmed by live-cell imaging (Fig. 5f; Video S4). In summary, NS1 tubules are dependent on cellular microtubules to aggregate toward the side of the nucleus.

### NS1 tubules are wrapped by vimentin to form aggresomes

**Fig 6:**
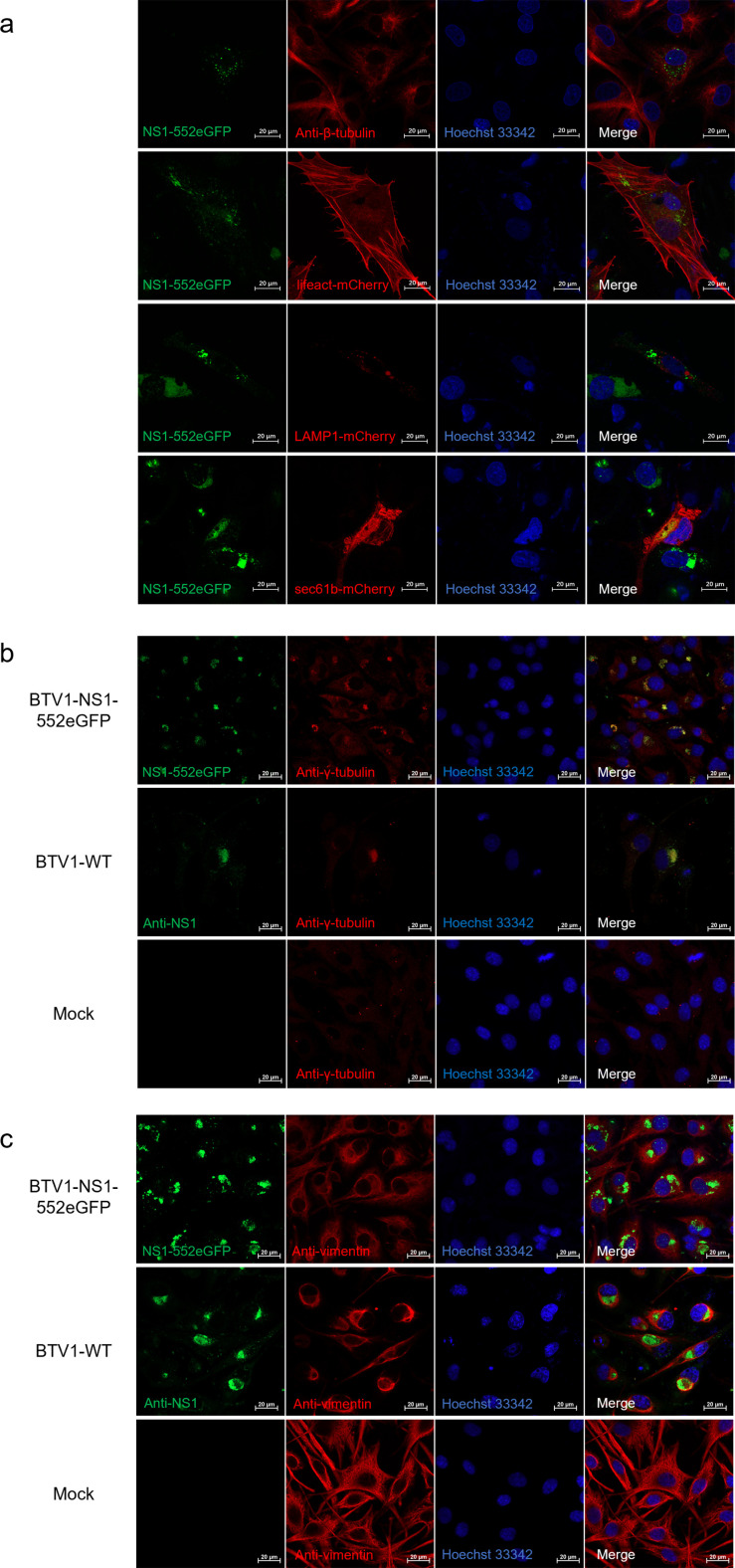
Subcellular localization and aggresome formation of NS1. (**a and b**) BSR cells were transfected with the lifeact-mCherry, sec61b-mCherry, and LAMP1-mCherry expression plasmids for 12 h, infected with BTV1-NS1-552eGFP (MOI = 0.1), fixed, and then infected with BTV1-NS1-552eGFP (MOI = 0.1) or BTV1-WT (MOI = 0.1) and probed with antibodies against β-tubulin and γ-tubulin to label cell microtubules and the MTOC (red). Hoechst 33342 staining (blue). Bar, 20 µm. (**c**) BSR cells were infected with BTV1-NS1-552eGFP (MOI = 0.1) or BTV1-WT (MOI = 0.1), fixed, and probed with antibodies against vimentin (red). The nuclei were stained with Hoechst 33342 (blue). Bar, 20 µm.

To determine the specific location of NS1 aggregates in infected BSR cells, cellular microtubules, microfilaments, endoplasmic reticula, lysosomes, and the MTOC were fluorescently labeled. BSR cells were infected with BTV1-NS1-552eGFP and the microtubules and MTOC were labeled using antibodies against β-tubulin and γ-tubulin, respectively, while the nuclei were labeled with Hoechst 33342 (Fig. 6a and b). The Lifeact-mCherry, sec61b-mCherry, and LAMP1-mCherry expression plasmids were used to label cellular microfilaments, endoplasmic reticula, and lysosomes of transfected BSR cells, respectively. After 12 h, cells were infected with BTV1-NS1-552eGFP, fixed, and labeled with Hoechst 33342 (Fig. 6a). The results showed that NS1 colocalized with the MTOC, but not the cellular microtubules, microfilaments, or lysosomes. The same results were obtained with BTV1-WT (Fig. 6b). When the production of aggregation-prone misfolded proteins exceeds the capacity of the proteasome, the proteins form an aggresome. The main features of the aggresome include dependence on microtubules, wrapping by a cage-like structure formed by vimentin, and colocalization with the MTOC in the perinuclear region of the cell (23–25). BSR cells infected with BTV1-NS1-552eGFP or BTV1-WT were fixed, incubated with antibodies against vimentin, and fluorescently labeled (Fig. 6c). The results showed that NS1 was wrapped by a cage-like structure formed by vimentin. In summary, NS1 localized to the MTOC and was wrapped by vimentin to form an aggresome.

## DISCUSSION

This work provides valuable evidence of the plasticity of the BTV genome and NS1 protein to accommodate and express exogenous genes. Three sites of BTV NS1 for insertion of exogenous genes were identified, and the recombinant viruses were successfully rescued. Importantly, after inserting tags at these three sites, recombinant NS1 exhibited different states in tubular and non-tubular forms. Deletion of the amino or carboxy terminus, or mutations of certain amino acids of NS1 could disrupt tubule formation (26). Insertion of a TC or eGFP tag between amino acids 156 and 157 also disrupted tubule formation, possibly by disrupting the hydrophobic groove along the head, thereby preventing embedding of the C-terminal arm. In contrast, insertion of the eGFP tag at the C-terminus of NS1 did not disrupt tubule formation as it is located on the surface of the tubule, allowing transport of larger peptide segments without disrupting the tubular structure (22, 27). NS1 tubules can be purified to lengths exceeding 1,000 nm, but vaccine particles with diameters of 10–100 nm are more favorable for enhancing vaccine efficacy (28). Interestingly, insertion of an eGFP tag between amino acids 534 and 535 did not disrupt tubule formation, but NS1 was more prevalent in the monomeric form, which could be attributed to eGFP interfering with NS1 monomer dimerization or intermonomer interactions within the layer, thereby shifting the equilibrium toward the monomeric state. NS1 tubules with chimeric immune antigens can carry thousands of antigen copies on the surface, effectively inducing both cellular and humoral immune responses, demonstrating potential as a new vaccine delivery system (29). The discovery of multiple insertion sites offers the possibility for a single NS1 monomer to carry multiple antigen epitopes, facilitating the development of multivalent vaccines. The successful rescue of these four recombinant viruses confirms the possibility of controlling the ratio conversion between the NS1 monomer and tubule forms, enhancing the potential of NS1 in vaccine delivery, therapy, and nanotechnology.

Recombinant viruses expressing fluorescent proteins with replication ability are powerful tools for basic and applied virology (30). Influenza virus-expressing fluorescence reporter proteins have been employed in imaging studies to examine the interaction between immune cells and virus-infected cells in the lungs of living mice (31). For quick antiviral drug screening, Mayaro, Ebinur Lake, and tick-borne encephalitis recombinant viruses with fluorescent proteins were created (32). The design of recombinant viruses expressing reporter genes will undoubtedly enhance our understanding of BTV (and related orbivirus) replication and pathogenesis. For example, a recombinant BTV1 expressing mCherry fused with NS3/NS3A was used for *in vivo* replication studies in *Culicoides sonorensis* and *Drosophila melanogaster*, demonstrating that *D. melanogaster* can serve as a model to study BTV replication and tropism in insects (33). Recently, NS1 of a recombinant BTV was fused to the NanoLuc luciferase reporter protein via a pig teschovirus-1 2A self-cleaving protease (34). This recombinant BTV enabled dynamic observation of viral spread to the spleen, thymus, and lungs during infection in a mouse model. We directly inserted eGFP or TC tags into NS1, constructing replicating fluorescent recombinant viruses to track NS1 in live cells. We found that NS1 does not initially fully aggregate on one side of the nucleus but undergoes a dynamic movement and aggregation process, which is dependent on cellular microtubules and the formation of NS1 tubules.

Early literature reported that NS1 is closely associated with the host cell cytoskeleton, but understanding of its biological functions remains limited (35). By labeling organelles, such as lysosomes, the endoplasmic reticula, and microfilaments, we observed that NS1 localized to the MTOC and was enveloped by a cage-like structure formed by vimentin. Vimentin interacts with BTV VP2, while NS1 remodels the vimentin network, suggesting that NS1 may play a role in the transport of BTV particles to the plasma membrane (36). Vimentin rearrangement facilitates the replication of various viruses, such as Japanese encephalitis virus and porcine reproductive and respiratory syndrome virus (PRRSV), which may provide a direction for NS1 to promote viral replication (37, 38). Additionally, NS1 also interacts with heat shock protein 90 (39). This led us to associate it with the concept of aggresomes, which accumulate at the MTOC via microtubule-mediated transport, as cellular responses to misfolded proteins are associated with a variety of diseases, such as Parkinson’s disease, Alzheimer’s disease, and Huntington’s disease, among other neurological disorders (40–42). Aggresomes selectively remove ubiquitinated or non-ubiquitinated misfolded proteins through the aggresome-autophagy pathway (43). Viral particles and proteins, as exogenous inclusion bodies, often aggregate in cells, leading to the formation of aggresomes, such as those formed by herpes simplex virus, PRRSV, and influenza virus, among others (44–46). Autophagy activated by infection plays a positive role in BTV replication (47). The aggresome formed by BTV NS1 may contribute to viral replication, providing new insights into the pathogenic mechanisms of BTV and facilitating the development of antiviral strategies.

Overall, we identified three distinct sites of BTV NS1 with a reverse genetics system that can accommodate large inserts of exogenous genes and successfully obtained recombinant viruses. We characterized the growth features, including plaque morphology, growth curves, and genetic stability, as well as the impact of exogenous gene insertion on the formation of NS1 tubules. Using confocal live-cell imaging, we observed in real-time the dynamic movement and aggregation of NS1 in BTV-infected cells, which is dependent on cellular microtubules and the formation of NS1 tubules. Ultimately, we confirmed that NS1 localizes to the MTOC and is enveloped by vimentin to form aggresomes. The tubular structures are characteristic of orbiviruses during replication in infected cells (48). This study contributes to our understanding of the role of tubules in orbivirus replication and provides a basis for the development of new vaccines based on the NS1 delivery system.

## MATERIALS AND METHODS

### Cells and viruses

BSR cells, a BHK-21 subclone, were cultured in Dulbecco’s modified Eagle’s medium (DMEM) supplemented with 5% fetal bovine serum (Sigma-Aldrich Corporation, St. Louis, MO, USA) at 37°C under an atmosphere of 5% CO_2_/95% air. Wild-type (WT) BTV serotype 1 (BTV1-WT), BTV1-NS1-156TC, BTV1-NS1-534eGFP, and BTV1-NS1-552eGFP were passaged in BSR cells, and viruses were titrated using the median tissue culture infectious dose (TCID_50_) assay.

### Plasmids and antibodies

The structure of BTV1 NS1 was predicted using AlphaFold 2 software and visualized with PyMol software (21, 49). Based on the predicted structure, the insertion site of the exogenous gene was determined. The genomic segments S1 to S10 of BTV1 (South African strain RSArrrr/01) were synthesized and cloned into the pUC57 vector under control of the T7 RNA polymerase promoter and restriction enzyme recognition sites (Beijing Tsingke Biotech Co., Ltd., Beijing, China). The eGFP gene was inserted into the corresponding S5 gene loci at specific positions within the NS1 protein (between amino acid residues 156 and 157, 534 and 535, as well as at the C-terminus). Additionally, a TC tag was introduced into the S5 gene locus between amino acid residues 156 and 157 of NS1. All mutant constructs were synthesized and cloned into the pUC57 vector, ensuring placement under control of the T7 RNA polymerase promoter and restriction enzyme recognition sites (Beijing Tsingke Biotech Co., Ltd.). Lifeact-mCherry (catalog no. 193300), sec61b-mCherry (catalog no. 90994), and LAMP1-mCherry (catalog no. 45147) were purchased from Addgene (Watertown, MA, USA).

All antibodies against BTV proteins were generated in our laboratory. Recombinant VP7, NS1, and NS2 proteins expressed in *Escherichia coli* were purified and used to produce specific polyclonal antibodies in rabbits. The anti-β-tubulin antibody was obtained from Abcam (Cambridge, UK). Antibodies against γ-tubulin and vimentin were purchased from Abways (Shanghai, China). The biarsenical dye FlAsH and fluorescently labeled secondary antibodies, including Alexa Fluor 488 and Alexa Fluor 568, were acquired from Invitrogen Corporation (Carlsbad, CA, USA).

### Virus recovery and purification

The plasmids, designed to carry a BTV genomic segment with a T7 promoter at the N-terminus and a restriction site (*Bsm*BI, *Bsa*I, or *Bbs*I) at the C-terminus, were expected to yield T7 transcripts through enzymatic linearization that are identical in sequence to the mRNA strand of the corresponding BTV genomic segment (16). The plasmid DNA was digested with the corresponding restriction enzymes at the 3′-terminus and purified by standard protocols. The digested plasmid DNA was used for RNA run-off transcription with an *in vitro* 5′-cap analog using the mMESSAGE mMACHINE T7 ULTRA Transcription Kit (Ambion, Inc., Austin, TX, USA). Synthesized RNA was purified with MicroSpin G-25 Columns (Cytiva, Marlborough, MA, USA), eluted, and stored at −80°C.

As described previously (50), BSR cell monolayers were initially transfected with 600 ng of RNA, comprising equimolar ratios of BTV segments encoding VP1, VP3, VP4, NS1, VP6, and NS2. Following an 18 h incubation period, a second transfection was performed using 600 ng of 10 distinct RNA segments in equimolar ratios. After 4 h, the transfection medium was aspirated and replaced with 1 mL of DMEM supplemented with 5% fetal bovine serum and 1% penicillin/streptomycin/fungizone. Transfection was carried out using Lipofectamine 2000 reagent (Invitrogen Corporation) in accordance with the manufacturer’s protocol. To rescue the recombinant virus, the S5 segment used in both transfection steps was replaced with transcripts derived from the corresponding mutant plasmid. Each recovered virus was plaque-purified, inoculated into monolayer BSR cells, and incubated at room temperature for 1 h. Subsequently, the cell monolayers were washed twice with phosphate-buffered saline (PBS) and overlaid with 1.5% low-melting point agarose (Invitrogen Corporation). After culturing the cell monolayers, single plaques were selected under a microscope and transferred into 0.5 mL of culture medium, which was then added to the monolayer BSR cells. Then, the above steps were repeated.

### Virus growth kinetics

BSR cell monolayers were simultaneously infected with BTV1-NS1-156TC, BTV1-NS1-534eGFP, BTV1-NS1-552eGFP, and BTV1-WT at a multiplicity of infection (MOI) of 0.1 and were subjected to TCID_50_ analysis at 12, 24, 36, 48, and 60 h post-infection. After three freeze/thaw cycles, the collected samples were stored at −80°C. The supernatants were then centrifuged for TCID_50_ analysis of BSR cells. As described previously (51), BTV1 S9 was amplified by reverse transcription polymerase chain reaction (RT-PCR) using specific primers. BSR cell monolayers were infected with BTV1-NS1-156eGFP and BTV1-WT at an MOI of 0.001, and samples were collected at 24, 48, 72, and 96 h post-infection. RNA was extracted from the samples, and the Ct values were determined. The copy number of the target gene in the sample was calculated in reference to a standard curve.

### Plaque assay

The virus was serially diluted by 10-fold in DMEM and added to the BSR cell monolayers cultured in 24-well culture plates. After incubation for 1 h at room temperature, the supernatant was discarded, and the cells were cultured for 72 h in medium containing 1.5% low-melting point agarose (Invitrogen Corporation). Plaques were stained with crystal violet and photographed. The plaque area was quantified with ImageJ software (https://imagej.net/ij/).

### Western blot analysis

Infected BSR cells were collected by centrifugation. Proteins were extracted, separated by electrophoresis, and electroblotted onto nitrocellulose membranes (Merck KGaA, Darmstadt, Germany), which were blocked with 5% skimmed milk in PBS and then probed with specific antibodies against the BTV VP7, NS2, and NS1 proteins, washed with PBST, followed by goat anti-rabbit horseradish peroxidase-conjugated immunoglobulin G (dilution, 1:10,000 dilution; Sigma-Aldrich Corporation), washed again with PBS, and developed with SuperSignal West Pico substrate (Thermo Fisher Scientific, Waltham, MA, USA).

### Electron microscopy

Virus-inoculated cells were scraped off with a cell scraper, collected by low-speed centrifugation (1,000 rpm), mixed with a special fixative for electron microscopy (Sigma-Aldrich Corporation), washed twice, fixed for 24 h, then fixed for 1.5 h with a 1% osmium solution, rinsed three times with 0.1 M phosphate buffer (pH 7.4), dehydrated with a series of graded ethanol solutions, embedded in epoxy resin, and cut into ultrathin sections, which were mounted on copper mesh, stained with a solution of hydrogen peroxide acetate and lead citrate, and finally examined and photographed under a Hitachi HT7700 transmission electron microscope (Hitachi Limited, Tokyo, Japan).

### Immunofluorescence

When using plasmids to label organelles, BSR cells were cultured in wells of cell culture plates (Corning, Inc., Corning, NY, USA) on cover glasses (Wuxi NEST Biotechnology Co., Ltd., Wuxi, China) for 12 h, transfected with the labeling plasmids, infected with the virus for 24 h, fixed in 4% paraformaldehyde, and osmotically treated with Triton X-100 (0.5%). The nuclei were stained with Hoechst 33342 (1 μg/mL) (Thermo Fisher Scientific) for 10 min and visualized with a confocal microscope. For antibody labeling of organelles or the NS1 protein, cells were initially treated with paraformaldehyde and Triton X-100. Following incubation with the primary antibody, the cover glasses were washed with PBS and subsequently incubated with goat anti-rabbit immunoglobulin G (Alexa Fluor 488 or 568; Abcam, Cambridge, MA, USA). As previously described (52), BTV1-NS1-156TC was fluorescently labeled using the TC-FlAsH II In-Cell Tetracysteine Tag Detection Kit (Thermo Fisher Scientific) in accordance with the manufacturer’s instructions.

### Live-cell imaging with confocal microscopy

To optimize imaging conditions, BSR cells were seeded at a low density in a glass-bottom dish (Wuxi NEST Biotechnology Co., Ltd.), cultured in an incubator for 12 h to ensure proper adhesion, and infected with BTV1-NS1-156eGFP, BTV1-NS1-534eGFP, or BTV1-NS1-552eGFP for 12 h. To visualize the cell nuclei, the culture medium was replaced with fresh medium supplemented with Hoechst 33342 (0.5 μg/mL) (Thermo Fisher Scientific), and the cells were incubated for 30 min. Fluorescence microscopy analysis at this stage confirmed successful nuclear labeling, while a subset of cells displayed green fluorescence, indicative of NS1-156eGFP, NS1-534eGFP, or NS1-552eGFP expression. To validate the role of cellular microtubules, the cells were treated with the inhibitor nocodazole (500 ng/mL), and the nuclei were stained with Hoechst 33342 (0.5 μg/mL). The glass bottom dish was then transferred to a live-cell imaging workstation, which was pre-equilibrated to maintain optimal culture conditions (5% CO_2_, 37°C). Cells that had just begun to exhibit or had not yet exhibited green fluorescence were selected, and appropriate fields of view were selected for live-cell imaging with an integrated confocal microscope. Live-cell imaging was performed using a laser scanning confocal microscope (ZEISS LSM 980 equipped with Airyscan 2; Carl Zeiss AG, Oberkochen, Germany) over a period of 12 to 24 h. Throughout the imaging process, cells were maintained under controlled conditions (5% CO_2_, 37°C), and images were acquired at 20 min intervals. The resulting image stacks and videos were subsequently processed and exported using ZEN 3.4 software (Carl Zeiss AG) for further analysis.

### Statistical analysis

Data analysis was performed with Prism 9 software (GraphPad Software, LLC, San Diego, CA, USA). Growth kinetics were analyzed using two-way analysis of variance (ANOVA) with the Holm-Šídák method. The copies (VP6) of BTV1-NS1-156eGFP and BTV1-WT were analyzed using two-way ANOVA with the Bonferroni’s multiple comparisons test. The plaque areas of BTV1-WT, BTV1-NS1-156TC, BTV1-NS1-534eGFP, and BTV1-NS1-552eGFP were analyzed using one-way ANOVA with Dunn’s multiple comparisons test. The fluorescence intensity of the background was measured using ZEN 3.4 software. The green fluorescent background of BTV1-NS1-156eGFP, BTV1-NS1-534eGFP, and BTV1-NS1-552eGFP was analyzed using one-way ANOVA with Tukey’s multiple comparisons test.

## Data Availability

All data have been included in the article and supplemental material. All other raw data supporting the conclusions of this study are available from the corresponding author upon reasonable request.

## SUPPLEMENTAL MATERIAL

Supplemental legends jvi.00896-25-s0001.docx Legends for Videos S1 to S4.

Video S1 jvi.00896-25-s0002.avi Live-cell imaging of BSR cells infected with BTV1-NS1-552eGFP.

Video S2 jvi.00896-25-s0003.avi Live-cell imaging of BSR cells infected with BTV1-NS1-534eGFP.

Video S3 jvi.00896-25-s0004.avi Live-cell imaging of BSR cells infected with BTV1-NS1-156eGFP.

Video S4 jvi.00896-25-s0005.avi Live-cell imaging of BSR cells infected with BTV1-NS1-552eGFP and the microtubule inhibitor nocodazole.

ASM does not own the copyrights to Supplemental Material that may be linked to, or accessed through, an article. The authors have granted ASM a non-exclusive, world-wide license to publish the Supplemental Material files. Please contact the corresponding author directly for reuse.
