# Supplementary material for: Visualization and tracking of tubule-derived, fluorescent-labeled NS1 as a marker of bluetongue virus in living cells
Source: J Virol. 2025 Aug 7;99(9):e00896-25. doi: 10.1128/jvi.00896-25 (PMC12456127; doi:10.1128/jvi.00896-25)
Supplement: Supplemental legends — Legends for Videos S1 to S4. [file jvi.00896-25-s0001.docx]

**Supplemental Movie** **Legends**

**Supplementary Video 1** BSR cells were infected BTV1-NS1-552eGFP for 12 h. Then, Live-cell imaging was utilized to observe it with images captured every 20 min. The nuclei were labeled with Hoechst 33342.

**Supplementary Video 2** BSR cells were infected BTV1-NS1-534eGFP for 12 h. Then, Live-cell imaging was utilized to observe it with images captured every 20 min. The nuclei were labeled with Hoechst 33342.

**Supplementary Video 3** BSR cells were infected BTV1-NS1-156eGFP for 12 h. Then, Live-cell imaging was utilized to observe it with images captured every 20 min. The nuclei were labeled with Hoechst 33342.

**Supplementary Video 4** BSR cells were infected BTV1-NS1-552eGFP for 12 h and then treated with the microtubule inhibitor nocodazole. Live-cell imaging was utilized to observe it with images captured every 20 min. The nuclei were labeled with Hoechst 33342.
